# Harnessing Dual Hydrogen Bonding and *Lewis* Acid–Base Interactions for Bio‐Inspired Symmetry‐Breaking Electrolytes in Aqueous Zinc‐Ion Batteries

**DOI:** 10.1002/anie.202516282

**Published:** 2025-08-31

**Authors:** Wei Zhang, Jie Chen, Chaohong Guan, Tianyun Qiu, Xiaodong Shi, Ruwei Chen, Zhenjing Jiang, Qingjin Fu, Xian Wu, Hang Yang, Mingqiang Liu, Peie Jiang, Yunpeng Zhong, Jianbin Zhou, Guanjie He

**Affiliations:** ^1^ Christopher Ingold Laboratory Department of Chemistry University College London London WC1H 0AJ UK; ^2^ School of Materials Science and Engineering Shanghai Jiao Tong University Shanghai 200240 P.R. China; ^3^ School of Photovoltaic and Renewable Energy Engineering University of New South Wales Sydney NSW 2052 Australia; ^4^ School of Chemistry and Chemical Engineering School of Marine Science and Engineering State Key Laboratory of Tropic Ocean Engineering Materials and Materials Evaluation Hainan University Haikou 570228 P.R. China; ^5^ Tsinghua Shenzhen International Graduate School Tsinghua University Shenzhen 518055 P.R. China; ^6^ Max Planck Institute for Sustainable Materials Max‐Planck‐Straße 1 40237 Düsseldorf Germany; ^7^ Key Laboratory of Precision and Intelligent Chemistry University of Science and Technology of China Hefei 230026 P.R. China

**Keywords:** Aqueous zinc‐ion batteries, Dual‐site hydrogen bonding, Hydrogen evolution suppression, *Lewis* acid–base interaction, Symmetry breaking

## Abstract

Aqueous zinc‐ion batteries (ZIBs) offer a safe, cost‐effective alternative for large‐scale energy storage but are hindered by zinc dendrite growth, hydrogen evolution reactions (HER), and unstable electrode–electrolyte interfaces. These challenges largely stem from strong dipole interactions between symmetric water molecules and Zn^2+^, which destabilize the electric double layer (EDL) and trigger parasitic reactions. Drawing inspiration from biological systems that use asymmetric molecular interactions to regulate aqueous environments, we introduce isobutyramide (IAM) as a multifunctional electrolyte additive. IAM features both carbonyl and amide groups, enabling it to act as a dual‐site hydrogen bond donor and acceptor. This disrupts the hydrogen‐bonding network in water, reduces water activity, and suppresses HER. Additionally, IAM's lone pairs coordinate strongly with Zn^2+^, restructuring the solvation sheath and mitigating uncontrolled Zn^2+^ migration that leads to dendrite formation. This dual‐function, symmetry‐breaking strategy stabilizes the EDL, enhances Zn plating/stripping reversibility, and suppresses interfacial degradation. Electrochemical tests confirm IAM's efficacy: Zn||Cu cells exhibit 99.68% Coulombic efficiency over 1,000 cycles, Zn||Zn symmetric cells remain stable for over 4,250 h, and full‐cell Zn||V_2_O_5_ and Zn||I_2_ systems show significantly enhanced cycling performance. Zn||I_2_ pouch cells also demonstrate robust long‐term operation. This bio‐inspired approach offers a scalable path to high‐performance, practical aqueous ZIBs.

## Graphical abstract

Bio‐inspired symmetry disruption in aqueous Zn‐ion batteries: Isobutyramide simultaneously breaks hydrogen‐bonding networks and coordinates Zn^2+^ ions, tailoring solvation structure and stabilizing Zn anodes. This molecular‐level electrolyte engineering suppresses dendrites, mitigates hydrogen evolution, and enables long‐lasting, high‐performance batteries.

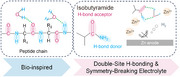

## Introduction

The transition to a low‐carbon energy future demands battery technologies that are safe, scalable, and environmentally sustainable.^[^ ^1^, ^2^, ^3^, ^4^, ^5^, ^6^, ^7^ ^]^ Among the contenders, aqueous zinc‐ion batteries (ZIBs) have emerged as promising candidates for large‐scale grid storage due to their inherent safety, cost‐effectiveness, and high theoretical capacity (820 mAh g^−1^ and 5851 mAh cm^−3^).^[^ ^8^, ^9^, ^10^, ^11^, ^12^ ^]^ Zinc metal's low redox potential and earth abundance make it an attractive anode material, while water‐based electrolytes offer superior ionic conductivity and fire resistance.^[^ ^13^, ^14^, ^15^ ^]^ However, practical applications remain limited by critical interfacial challenges including zinc dendrite growth,^[^ ^16^, ^17^, ^18^ ^]^ parasitic hydrogen evolution reactions (HER),^[^ ^19^, ^20^, ^21^, ^22^, ^23^, ^24^ ^]^ and unstable electrode–electrolyte interfaces.^[^ ^25^, ^26^, ^27^, ^28^, ^29^, ^30^ ^]^ These issues fundamentally stem from the molecular structure and dynamic behavior of water‐based electrolytes, particularly the highly symmetric arrangement of water molecules and sulfate ions that govern solvation and surface phenomena.^[^ ^14^, ^31^, ^32^, ^33^ ^]^

**Scheme 1:**
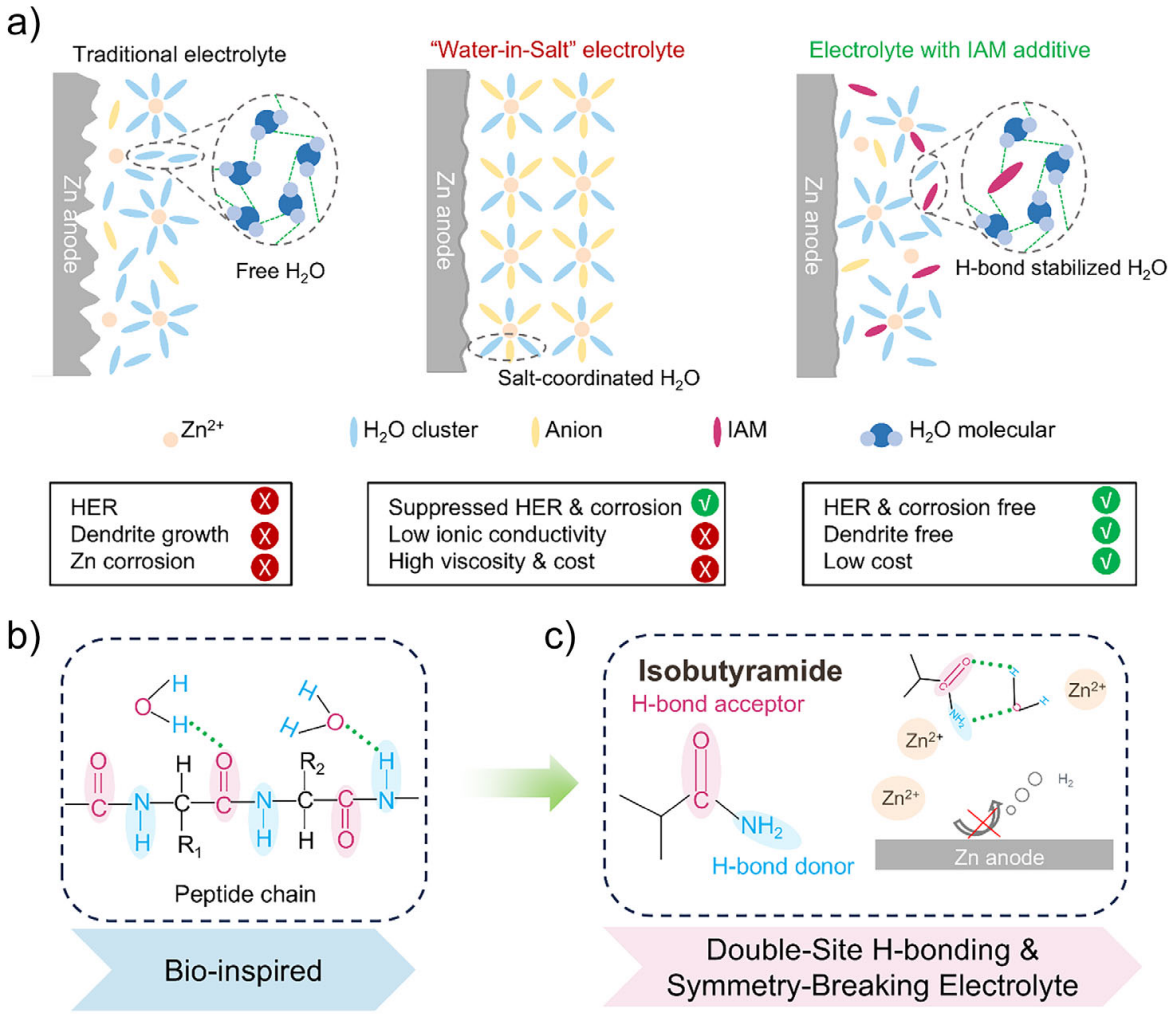
Schematic illustration of the electrolyte design philosophy of this work.

In conventional dilute ZnSO_4_ electrolytes, Zn^2+^ ions are tightly coordinated by six water molecules, forming an octahedral Zn(H_2_O)_6_^2+^ solvation shell.^[^ ^34^, ^35^ ^]^ This highly symmetric solvation structure is further enveloped by a hydrogen‐bonded network of water and sulfate anions.^[^ ^36^ ^]^ At the electrode interface, water molecules orient their dipoles to form a compact electric double layer (EDL), whose symmetry and stability critically influence Zn^2+^ ion transport and electrodeposition.^[^ ^36^ ^]^ However, this symmetric configuration fosters several detrimental effects.^[^ ^37^ ^]^ The strong dipole interactions and uniform hydrogen bonding enhance proton mobility through the *Grotthuss* mechanism, accelerating HER.^[^ ^38^ ^]^ Simultaneously, uniform dipole alignment leads to spatially uneven zinc nucleation and growth, manifesting as dendrites that compromise battery safety and longevity (Scheme 1a).^[^ ^20^, ^39^, ^40^ ^]^

Addressing these challenges requires more than simply adjusting electrolyte concentration (e.g., “*water‐in‐salt*”)^[^ ^41^, ^42^, ^43^ ^]^ or adding co‐solvents.^[^ ^44^, ^45^, ^46^ ^]^ Instead, it demands a fundamental reconfiguration of solvation and interfacial structures at the molecular level. Here, the concept of symmetry breaking provides an insightful framework.^[^ ^47^ ^]^ Rooted in *Landau's* theory of phase transitions,^[^ ^48^ ^]^ symmetry breaking describes how a system transitions from a high‐symmetry state to a lower‐symmetry state under certain conditions, resulting in emergent properties and novel phases. This paradigm explains a vast array of phenomena in condensed matter physics, from crystallization to magnetism, by revealing how subtle structural perturbations can dramatically alter macroscopic behavior.

Translating this principle to aqueous electrolytes, disrupting the intrinsic symmetry of water and its interactions with ions can lead to fundamentally altered solvation dynamics and interfacial configurations.^[^ ^47^ ^]^ Such symmetry disruption breaks the continuity of the hydrogen bonding network, rearranges ion coordination environments, and modifies the electric double layer structure, collectively suppressing HER and dendrite formation.^[^ ^36^ ^]^ While symmetry breaking is a well‐studied concept in physics and materials science, its application in electrochemical electrolyte design remains nascent.^[^ ^49^, ^50^ ^]^

Nature, however, offers a remarkable example of how asymmetric molecular interactions govern aqueous environments with exquisite precision.^[^ ^38^ ^]^ Biological systems rely on complex networks of asymmetric hydrogen bonds and metal–ligand coordination to regulate water structure, ion transport, and catalytic activity (Scheme 1b). For instance, peptide bonds in proteins act simultaneously as hydrogen bond donors and acceptors, creating dynamic, directionally biased hydration shells.^[^ ^51^ ^]^ Metalloproteins coordinate metal ions such as Zn^2+^ through *Lewis* acid–base interactions with nitrogen and oxygen atoms, enabling fine control over reactivity and transport.^[^ ^33^, ^52^ ^]^ These asymmetric interactions result in stable yet flexible aqueous microenvironments, achieving functions that synthetic systems strive to emulate.^[^ ^53^ ^]^

Inspired by biomimetic principles, we introduce isobutyramide (IAM) as a novel electrolyte additive featuring dual‐site functionalities capable of simultaneously disrupting water's symmetric hydrogen‐bonding network and coordinating with Zn^2+^ ions (Scheme 1c). IAM is a low‐cost and readily available organic molecule that incorporates both amide and carbonyl groups within a compact molecular framework. Its small molecular weight and excellent water solubility make it highly suitable for aqueous systems, ensuring minimal impact on viscosity and ionic conductivity. The amide group in IAM acts as both a hydrogen bond donor and acceptor, effectively disturbing the structured hydrogen‐bond network of bulk water and reducing its activity—thereby suppressing the HER. Simultaneously, the carbonyl oxygen serves as a *Lewis* base, coordinating with Zn^2+^ to restructure the solvation sheath and limit ion diffusion, which is crucial for suppressing dendritic growth. This dual‐interaction, symmetry‐breaking design allows IAM to induce a substantial reorganization of both the bulk electrolyte and the interfacial EDL, resulting in significantly enhanced Zn plating/stripping reversibility and overall interfacial stability. Unlike many previously reported electrolyte additives that are designed to address a single issue such as hydrogen evolution suppression or dendrite control, IAM combines dual‐site hydrogen bonding capability with strong *Lewis* base coordination in a single, compact molecular structure. This multifunctional design enables simultaneous disruption of the hydrogen‐bonding network in bulk water and reconfiguration of Zn^2+^ solvation structures, providing synergistic regulation of both electrolyte structure and interfacial stability.

The effectiveness of this symmetry‐breaking electrolyte design is reflected in its outstanding electrochemical performance across multiple cell configurations. Zn||Cu cells cycled at 1 mA cm^−2^ achieve a high average Coulombic efficiency of 99.68% over 1000 cycles, indicating suppressed side reactions and highly reversible Zn plating/stripping. Zn||Zn symmetric cells exhibit exceptional stability, operating for over 4250 h at 1 mA cm^−2^ without short‐circuiting, underscoring effective dendrite suppression and interfacial robustness. In full‐cell systems, Zn||I_2_ cells display excellent cycliability over 10 000 cycles at 2 A g^−1^ in coin cell format, while scaled pouch cells maintain stable cycling under practical current densities—demonstrating the additive's scalability and real‐world applicability. Moreover, the IAM‐enhanced electrolyte significantly improves the performance of Zn||V_2_O_5_ cells, delivering superior capacity retention and prolonged cycling stability, further validating the broad applicability of this bio‐inspired design strategy across diverse cathode chemistries. Molecular dynamics simulations and spectroscopic analyses confirm that IAM induces the formation of an asymmetric solvation sheath and modifies the interfacial EDL structure, validating the proposed dual‐site symmetry‐breaking mechanism. This bio‐inspired approach provides a transformative electrolyte engineering paradigm that redefines solvation and interface chemistry through symmetry disruption, offering a powerful pathway to realize safe, durable, and high‐performance aqueous ZIBs.

## Results and Discussion

### Intermolecular Interactions

When designing electrolyte additives for aqueous zinc‐ion batteries, molecular geometry, functional group polarity, and solvation behavior are critical for optimizing interfacial stability and ionic transport.^[^ ^39^ ^]^ Isobutyramide (IAM), a compact organic molecule with an inherently asymmetric structure, presents a unique combination of functional groups capable of dual interaction modes with the electrolyte environment. Its branched alkyl backbone alongside spatially separated carbonyl (C═O) and amide (─NH_2_) groups (Scheme 1c) creates an uneven electron distribution, effectively breaking the symmetric hydrogen bonding network of bulk water.^[^ ^54^ ^]^ The carbonyl oxygen acts as a *Lewis* base, coordinating with Zn^2+^ ions, and modifying their solvation sheath, while the amide group simultaneously functions as both a hydrogen bond donor and acceptor, disrupting the continuity of the water hydrogen‐bonding network. This asymmetry promotes the formation of a more heterogeneous and ordered solvation environment near the electrode interface, which contributes to a stabilized electric double layer (EDL), suppresses hydrogen evolution reactions, and mitigates dendrite growth. Compared to symmetric solvent molecules, IAM's molecular asymmetry facilitates more complex intermolecular interactions and dynamic restructuring of the electrolyte interface.^[^ ^49^, ^50^ ^]^ Subsequent spectroscopic, simulation, and electrochemical analyses further elucidate how IAM reshapes Zn^2+^ coordination and EDL composition, enhancing zinc plating/stripping reversibility and battery longevity.

**Figure 1:**
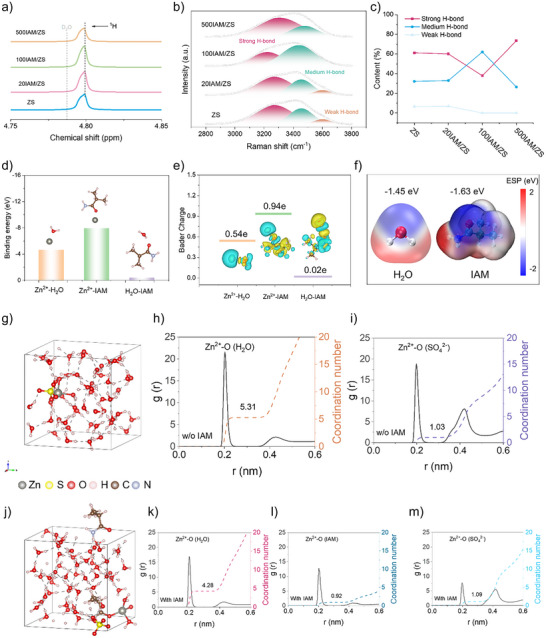
Solvation structure analysis. a) ^1^H NMR spectra of the electrolyte with and without IAM (solvent: D_2_O), showing changes in the proton environment upon IAM addition. b) Fitted Raman spectra in the O─H stretching region. c) Ratio trends of strong, medium, and weak hydrogen bonds, indicating modulation of the hydrogen‐bonding network. d) Binding energies of Zn^2+^─H_2_O, Zn^2+^─IAM, and H_2_O─IAM complexes. e) Charge density difference maps and electron donation analysis for the same complexes. f) Electrostatic potential mappings of H_2_O and IAM. g) and j) Enlarged 3D snapshots of Zn^2+^ solvation structures from AIMD simulations of the pure and IAM‐containing electrolytes. h) and i) Radial distribution functions (RDFs) and coordination numbers (CNs) for Zn^2+^─O(H_2_O) and Zn^2+^─O(SO_4_^2−^) in the pure electrolyte. k)–m) RDFs and CNs for Zn^2+^─O(H_2_O), Zn^2+^─O(IAM), and Zn^2+^─O(SO_4_^2−^) in the IAM‐containing electrolyte.

First, nuclear magnetic resonance (NMR), Raman spectra, and Fourier transform infrared (FT‐IR) spectra were performed to study the intermolecular interactions in various electrolyte systems. Every prepared electrolyte in Figure S1 is transparent and free of precipitates (ZnSO_4_ denoted as ZS; 20IAM/ZS, 100IAM/ZS, and 500IAM/ZS represent ZS electrolytes containing 20, 100, and 500 mM IAM, respectively). The ^1^H peak of D_2_O is located at 4.787, as seen in Figure 1a. The peak positively moves to 4.800 ppm upon adding zinc salt. Given the tight coordination between D_2_O and Zn^2+^, this might be explained by a weaker proton shielding and a decreased surrounding electron density in H_2_O molecules, which would indicate less free water in the Zn salt environment. When the amount of IAM additives increases, this peak value shifts downward, indicating that more free H_2_O molecules are eliminated. This result also demonstrates that IAM molecules have the ability to significantly interact with H_2_O, reduce the interaction between Zn^2+^ and water molecules, and break and reorganize the hydrogen bond network—all of which are beneficial for inhibiting the hydrogen evolution reaction (HER).^[^ ^31^ ^]^ When the concentration of IAM exceeds 0.1 M, as seen in Figure S2, some new peaks appear at around 770, 1450, and 2980 cm^−1^. These new peaks are thought to be the result of the chelation of the IAM molecule and Zn^2+^ ions. The *v*‐SO_4_^2−^ band should be associated with an extra peak located approximately at 980 cm^−1^. Such a peak can be deconvoluted into two distinct ion pairs (CIP: contact ion pair; Figure S3), according to *Eigen–Tamm* theory.^[^ ^55^, ^56^ ^]^ SSIP stands for solvent‐separated ion pair. As seen in Figure S3a, as IAM concentrations increase, this peak shifts to lower frequencies and the CIP ratio decreases. This suggests that IAM molecules can enter the CIP and replace the SO_4_^2−^ group, which can prevent the formation of the infamous Zn_4_SO_4_(OH)_6_·*x*H_2_O.^[^ ^54^ ^]^ The ratio of strong H‐bonds shows an upward trend in Figure 1b,c as the contents of IAM additives increase, while those of other H‐bonds (i.e., medium and weak) decrease. This suggests that IAM can strengthen the H‐bond network among free water molecules and prevent parasitic reactions. IAM strengthens the O─H bond in H_2_O, as seen by the positive shift in the O─H bending and stretching vibrations of the FT‐IR spectra shown in Figure S4a,b as the IAM concentration increases. This tendency is further supported by the shift in SO_4_^2+^’s vibration stretching observed in Figure S4c.^[^ ^57^ ^]^

Furthermore, Zn^2+^, IAM, and H_2_O molecule intermolecular interactions were studied using quantum chemistry computations. It is clear from Figure 1d that Zn^2+^ ions are more likely to bond with IAM molecules since their binding energy is significantly more negative for Zn^2+^‐IAM than it is for Zn^2+^‐H_2_O. Metal cations’ solvation structure has been altered, which is typically shown by a changed binding energy of metal cations with other molecules.^[^ ^58^ ^]^ Three structures (Zn^2+^‐IAM, Zn^2+^‐H_2_O, and H_2_O‐IAM; Figure 1e) were subjected to the Bader analysis in order to provide an intuitive comparison. Zn^2+^ donates more electrons to IAM (0.94 e) than it does to H_2_O (0.54 e). The results validate the preferred binding of Zn^2+^ with IAM once more. IAM's preferential binding of Zn^2+^ in the solvation sheath is further supported by the electrostatic potential (ESP) mapping of IAM, which is shown in Figure 1f and displays a stronger local electronegativity (−1.63 eV) than H_2_O (−1.45 eV).^[^ ^57^ ^]^ It is also notable that H_2_O interacts with IAM, as shown by the electron donation (0.02 e, Figure 1e) and binding energy (−0.41 eV, Figure 1d). Hydrogen bonds between O─H of H_2_O and C═O of IAM (Figure S5a) or H─O of H_2_O and NH_2_ of IAM (Figure S5b) enable the binding of H_2_O‐IAM.

Afterward, Zn^2+^ solvation structures were further investigated using ab initio molecular dynamics (AIMD) simulations.^[^ ^59^ ^]^ Consistent with earlier research,^[^ ^58^, ^60^ ^]^ the statistical findings in Figures 1g and S6 show five H_2_O and one SO_4_^2−^ in the Zn^2+^ primary solvation shell (PSS) in the pure electrolyte. On the other hand, the IAM‐containing system shown in Figure 1j has a novel Zn^2+^ solvation structure made up of IAM, H_2_O molecules, and SO_4_^2−^, indicating that IAM may chelate with Zn^2+^ and disrupt the PSS. Some H_2_O molecules in the Zn^2+^ solvation structure can be replaced by the carbonyl functional group in IAM molecules, not the amino functional groups.

Additionally, coordination number (CN) and radial distribution functions (RDFs) analysis were carried out. A distinct peak of Zn^2+^‐O in pure electrolyte was discovered in Figure 1h,i, about 2 Å distant from Zn^2+^, which corresponds to H_2_O and SO_4_^2−^ in PSS. Similarly, the ∼2 Å sharp peaks from H_2_O, SO_4_^2−^, and IAM can also be seen in the electrolyte containing IAM. These peaks indicate that some IAM molecules enter and stay stable in the PSS of Zn^2+^.^[^ ^46^, ^58^, ^60^ ^]^ Furthermore, it is evident that in the first hydration layer of the IAM‐containing electrolyte, the average CNs of Zn^2+^‐O (H_2_O; Figure 1k), and Zn^2+^‐O (IAM; Figure 1l), and Zn^2+^‐O (SO_4_^2−^; Figure 1m) are about 4.28, 0.92, and 1.09 (i.e., [Zn(H_2_O)_4.28_IAM_0.92_(SO_4_)_1.09_]), respectively. On the other hand, in the pristine situation, the average CN of Zn^2+^‐O (H_2_O; Figure 1h) and Zn^2+^‐O (SO_4_^2−^; Figure 1i) is 5.31 and 1.03. This outcome shows that the amount of solvated H_2_O molecules inside Zn^2+^ PSS is actually tuned by adding IAM molecules, going from 5.31 in the pure electrolyte to 4.28 in the electrolyte system including IAM. In addition, Zn||Cu cells were made to verify the ideal IAM content. The battery with 100 mM IAM additives has the greatest average Coulombic efficiency (99.1%) in Figure S7, outperforming two other groups (20 mM IAM: 98.2%; 500 mM IAM: 97.5%). Hence, in this experiment, the electrolyte containing 100 mM IAM is the optimized group (designated as DE; 1 M ZnSO_4_ is designated as BE).

### Corrosion Inhibition

**Figure 2:**
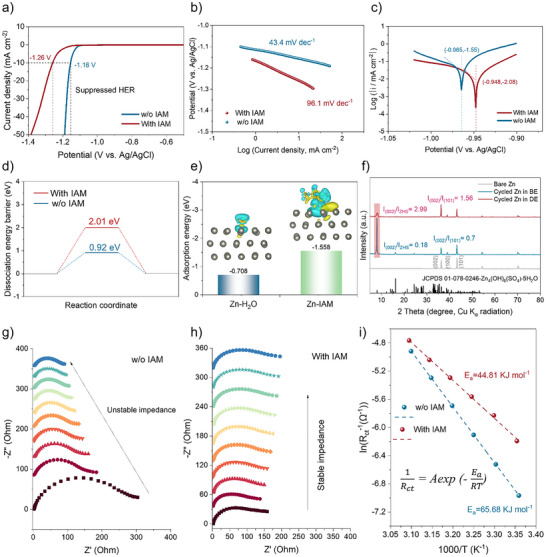
Corrosion inhibition. a) Linear sweep voltammetry (LSV) curves showing the HER behavior of electrolytes with and without IAM additives. b) Corresponding Tafel slope curves. c) Linear polarization curves of Zn anodes in the presence and absence of IAM additives. d) Calculated dissociation energy barriers of H_2_O in both electrolytes. e) Adsorption energies of H_2_O and IAM molecules on Zn (002) surfaces (insets: charge density difference plots showing spatial charge redistribution upon adsorption, with charge depletion and accumulation represented by cyan and yellow regions, respectively). f) GIXRD patterns of Zn surfaces after cycling. g) and h) In situ EIS spectra of Zn||Zn symmetric cells without and with IAM additives. i) Arrhenius plots and corresponding activation energies of Zn||Zn symmetric cells operated in electrolytes with and without IAM additives at various temperatures.

In addition to altering the Zn^2+^ solvation structure and reconstructing the water H‐bonding network, the impact of IAM on the electrolyte/electrode interface, may have a significant effect on Zn anode electrochemical performance.^[^ ^29^ ^]^ To study the anticorrosion capacity and interfacial chemistry of zinc anodes under electrolytes containing IAM additions, a variety of electrochemical tests were performed. Initially, we used a Zn||Ti configuration and a linear sweep voltammetry (LSV) method against an Ag/AgCl reference electrode. Figure 2a shows that the hydrogen evolution reaction (HER) starting potential on the anode side could be left shifted by the IAM additive, suggesting that HER was hindered in DE compared to BE. Additionally, supporting this same finding are the Tafel slopes (Figure 2b), which show an increase from 43.4 mV dec^−1^ in the pure electrolyte without IAM to 96.1 mV dec^−1^ in the electrolyte with IAM. The HER rate is lowered as a result of IAM addition, as indicated by the Tafel slope raising.^[^ ^57^ ^]^

Besides, the linear polarization curves are shown in Figure 2c. “Tafel Extrapolation” is the most basic method for assessing corrosion current density experimentally. In Figure 2c, the corrosion potential was clearly visible at the bottom of the Tafel plot. A line that was drawn perpendicular to the Potential (*x)*‐ axis was then drawn. At a certain point on the aforementioned perpendicular line, the extrapolation lines of the anodic and cathodic linear sections will cross. The corrosion current density may be computed from the *y*‐axis value at this junction location. When IAM additives are added, the corrosion current density decreases together with a positive shift in the corrosion potential from −0.965 to −0.948 V. This suggests suppressed corrosion and promotes enhanced electrochemical performances. The alteration in the energy barrier of H_2_O dissociation brought on by IAM may account for the reduced HER. Zn surface is used to model the dissociation of H_2_O because electrochemical reactions usually occur at the interface between the electrolyte and electrode and entail the transfer of electrons. In ZnSO_4_‐H_2_O‐IAM, the energy barrier of H_2_O dissociation is 2.01 eV, which is much higher than that of ZnSO_4_‐H_2_O (0.92 eV), as per Figure 2d. Consequently, Zn in ZnSO_4_‐H_2_O ‐IAM significantly inhibits HER.

In addition, calculations are made about the adsorption of various solvent molecules on the zinc metal surface. The contact between Zn and IAM (H_2_O) clearly exhibits charge transfer, as seen in Figure 2e. We computed the specific absorption energies of IAM and H_2_O at various Zn surface locations.^[^ ^57^ ^]^ It is seen that there is not much interaction between Zn and H_2_O at various places (Figure S8). With an absorption energy of −0.708 eV, the most stable one is arranged in a bridge configuration (Figure 2e). On the other hand, regardless of the position (top, bridge, hollow, or parallel on the Zn surface) that is used in the computation, the interaction between Zn and IAM is substantially greater. At a top/bridge/hollow location, the adsorption energy of IAM on Zn may reach −1.558 eV.

Moreover, the cycled Zn foils in various electrolytes were investigated using grazing incidence X‐ray diffraction (GIXRD), which is a trustworthy method to detect the texturing and orientation anisotropy of thin films. The Zn dendrite growth is negatively impacted by the intensity ratio of (002) to (101) peaks of the cycled zinc anodes in BE, as shown in Figure 2f, which was measured to be 0.70. This value corresponds to the (101)‐dominating plane.^[^ ^61^ ^]^ With the help of IAM, this value rises to 1.56, indicating the dominating (002) facet development; as a result, compact Zn deposition and reduced Zn dendrites may be achieved.^[^ ^61^ ^]^ Notably, IAM is also helpful in preventing the development of the notorious Zn_4_SO_4_(OH)_6_·5H_2_O (ZHS), as seen by its corresponding much decreased intensity (*I*_(002)_/*I*_ZHS_: 0.18 in BE versus 2.99; Figure 2f).

Additionally, we used an ordered discharging and standing procedure using Zn||Zn symmetric cells to perform an in situ electrochemical impedance spectroscopy (EIS) approach to examine the structural stability of the Zn anode/electrolyte interface following zinc electrodeposition. The charge transfer impedance of the one using BE clearly fluctuates with the test time, as shown in Figure 2g,h. In contrast, the other one with IAM additives exhibits a stable impedance, further confirming the ability of the adsorbed IAM to stabilize the interface. Based on the traditional *Arrhenius* equation (the inset of Figure 2i), the activation energy (*E_a_*) using the EIS technique under different temperatures was used to further determine the enhanced interphase chemistry between the Zn anode and electrolyte. With IAM additions, it is simple to observe that *E_a_* decreases from 65.68 to 44.81 kJ mol^−1^, confirming once more the enhanced interfacial redox kinetics in the electrolyte containing IAM.^[^ ^62^ ^]^

### Stripping/Plating Morphologies

**Figure 3:**
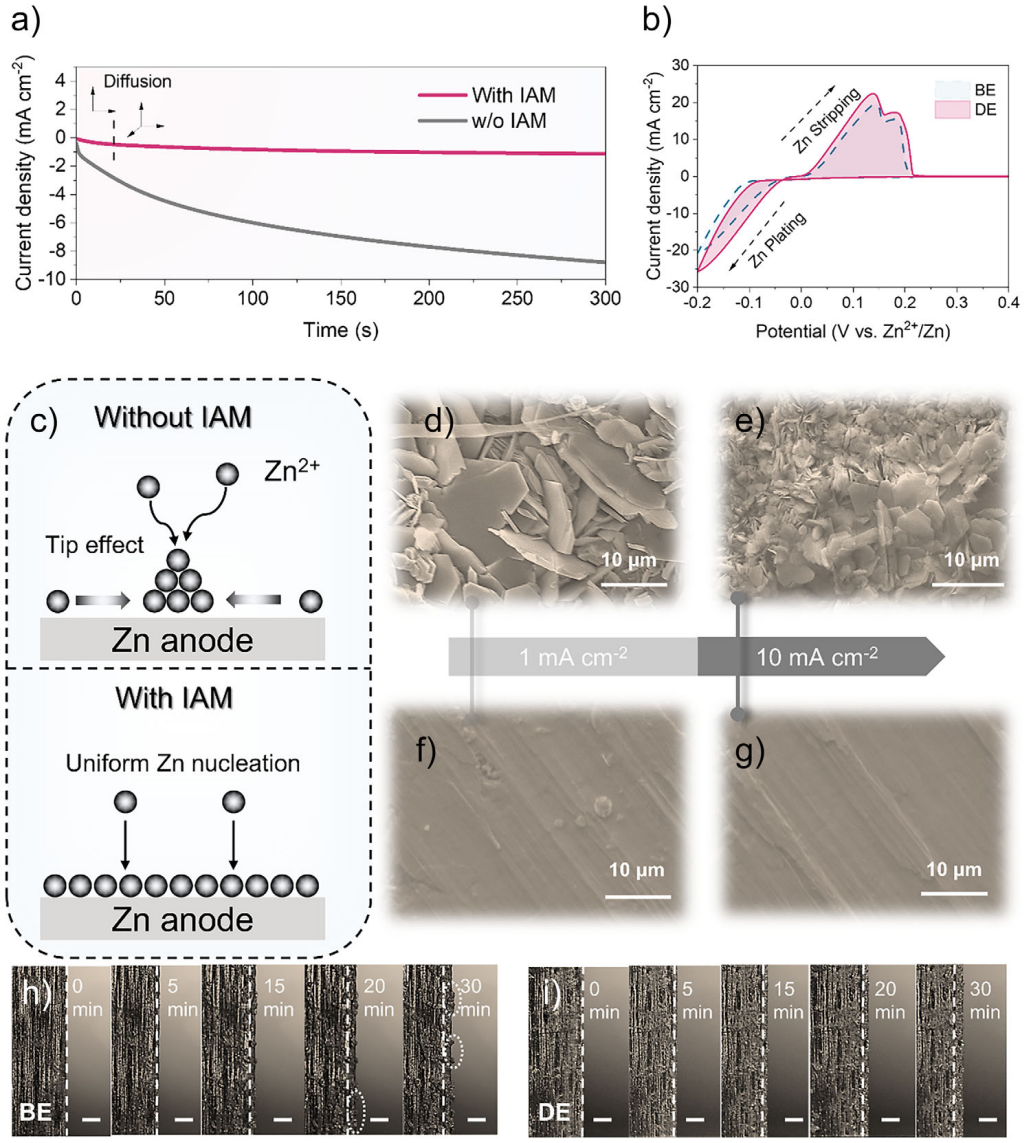
a). Chronoamperometry (CA) curves of Zn||Zn symmetric cells in electrolytes with and without IAM additives at −150 mV. b) Cyclic voltammetry (CV) curves of Zn||Cu asymmetric cells under the same electrolyte conditions. c) Schematic illustration of Zn^2+^ diffusion and nucleation behavior on Zn surfaces in the presence and absence of IAM additives. d) and e) SEM images of cycled Zn electrode surfaces using the baseline electrolyte (BE, 1 M ZnSO_4_). f) and g) SEM images of cycled Zn electrode surfaces using the IAM‐containing electrolyte (DE, 1 M ZnSO_4_+100 mM IAM). h) and i) In situ optical microscopy images (scale bar: 300 µm) showing Zn deposition morphology at 10 mA cm^−2^ in electrolytes without and with IAM additive, respectively.

The nucleation behavior was investigated using a chronoamperometry (CA) test at a specified overpotential (−150 mV) in order to examine the effects of various electrolytes on Zn deposition behavior. As can be seen in Figure 3a, there is a lengthy and uncontrolled 2D diffusion process shown in the current density's constant reduction within 300 s when using BE.^[^ ^58^ ^]^ Zn^2+^ diffuses to the tips in this planar diffusion mechanism, increasing the formation of Zn dendrites over time (Figure 3c). The Zn anode with DE, on the other hand, shows stable current density, indicating 3D diffusion‐controlled nucleation. As a result of the increased uniform nucleation sites, this behavior can effectively reduce Zn dendrite development. To further investigate the impact of the IAM‐containing electrolyte on the early nucleation behavior, cyclic voltammetry (CV) curves of Zn^2+^ plating/stripping on Cu foil were subsequently carried out. The cell that uses DE clearly has a smaller nucleation overpotential than the one that uses BE (Figure 3b). As demonstrated by the morphology of plated Zn, the decreased nucleation overpotential promotes the development of homogenous Zn nucleation sites.

The performance of batteries is influenced by the morphologies of zinc deposits. To explore the role of IAM on zinc electrodeposition behaviors at 1 and 10 mA cm^−2^ with 1 mAh cm^−2^, we further utilized scanning electron microscopy (SEM). All of the zinc deposits in Figure 3d,e showed quasi‐hexagonal platelets, even though some of the zinc showed scattered platelets when the current density hit 10 mA cm^−2^. Because of the Zn(002) slab's low thermodynamic free energy, these morphologies correspond well with earlier findings of aqueous ZnSO_4_ electrolytes.^[^ ^27^ ^]^ Furthermore, it should be highlighted that, in line with other research, Zn nucleation size reduces and Zn deposits get denser as current density rises. Previous studies have confirmed that the nuclei size is inversely proportional to the overpotential, while an increase in current density results in a higher nucleation overpotential, thereby explaining this phenomenon.^[^ ^63^, ^64^ ^]^ On the other hand, the Zn deposits in the electrolyte with IAM additions at different current densities in Figure 3f,g exhibit smoother and denser plane‐like surfaces. These results correspond well with Figure 3a–c. In addition, we used in situ optical microscopy to investigate the behaviors of zinc deposition at 10 mA cm^−2^. Figure 3h shows that, only after 30 min, the Zn dendrites in BE became progressively bigger on the Zn surface in an uneven and loose way. On the other hand, under the same conditions, the deposited Zn in Figure 3i displayed denser and flatter morphologies thanks to the use of electrolytes containing IAM.

The aforementioned findings thus support the idea that IAM molecules may significantly smooth the zinc surface and inhibit the formation of zinc dendrites.

### Battery Performance

**Figure 4:**
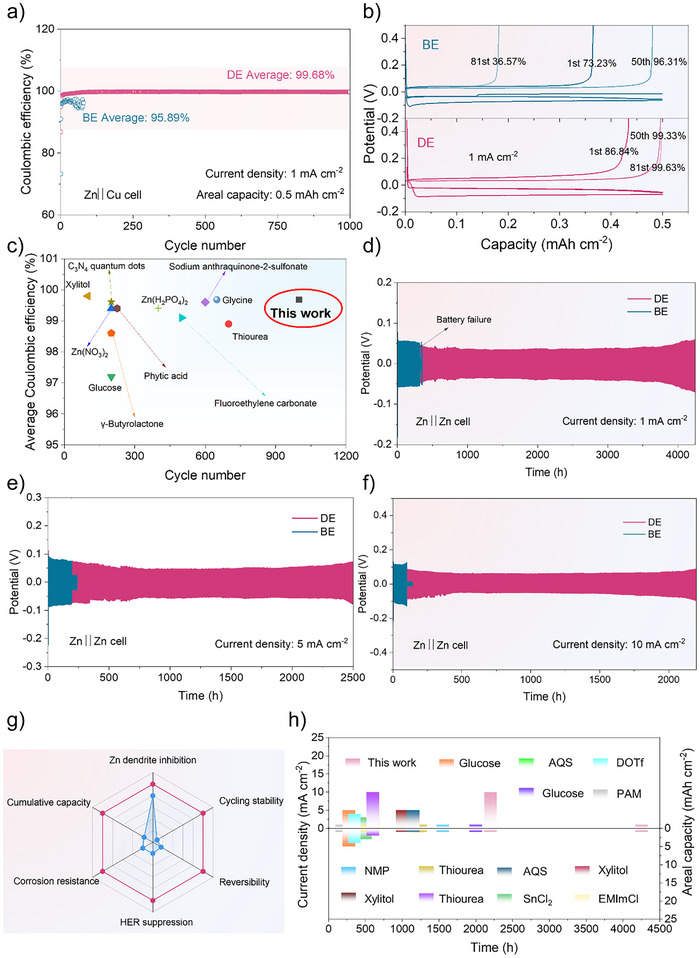
a). Coulombic efficiencies (CEs) and b) charge/discharge curves of Zn||Cu cells in electrolytes with and without IAM additives. c) Comparison of cycling stability and average CE of symmetric Zn||Zn cells using various reported electrolyte additives. d)–f) Electrochemical performance of Zn||Zn symmetric cells at 1, 5, and 10 mA cm^−2^, respectively, with a fixed areal capacity of 1 mAh cm^−2^. g) Performance comparison between IAM‐containing electrolyte (DE, red, 1 M ZnSO_4_+100 mM IAM) and baseline electrolyte (BE, blue, 1 M ZnSO_4_). h) Battery performance benchmarking of this work against previously reported additives, including sodium anthraquinone‐2‐sulfonate (AQS), *N,N*‐dimethylformamidium trifluoromethanesulfonate (DOTf), polyacrylamide (PAM), *N*‐methyl pyrrolidone (NMP), and 1‐ethyl‐3‐methylimidazolium chloride (EMImCl).

The aforementioned tests and theoretical investigations showed how beneficial IAM additives are in reducing Zn dendrite development and preventing adverse effects. Zn||Cu, Zn||Zn, and full cells with varying cathodes were also created in order to assess zinc deposition behaviors with IAM. To start, Figure 4a,b demonstrate that the Zn||Cu cell using BE displayed a low initial Coulombic efficiency (CE), which is indicative of an inferior initial Zn lattice fitting stage on the Cu substrate. The battery can only operate around 80 cycles without protection, with a poor average CE of 95.89% because of to the previously described adverse reactions and dendritic development. In comparison, the Zn||Cu cell with IAM additives may provide an enhanced initial CE of 86.84% and a high average CE of 99.68% over 1000 cycles, exceeding the performance of many documented electrolyte additive studies (Figure 4c).^[^ ^54^, ^58^, ^62^, ^65^, ^66^, ^67^, ^68^, ^69^, ^70^, ^71^, ^72^, ^73^, ^74^ ^]^

This outcome once again demonstrated the benefits of IAM additives in enhancing Zn stripping and plating behaviors’ reversibility. Moreover, Figure 4d–f show extended battery lifespans: IAM‐added Zn||Zn cells can operate continuously for 4250, 2500, and 2200 h at 1, 5, and 10 mA cm^−2^, respectively. But under the same circumstances, the Zn||Zn symmetric cells employing BE can only cycle for 314 hours, 193, and 100 h, respectively. As demonstrated in Table S1 and Figure 4h, such excellent cycling performance results in an exceptional cumulative capacity of 11 000 mAh cm^−2^, surpassing that of the majority of reported electrolyte additive works.^[^ ^57^, ^58^, ^66^, ^70^, ^71^, ^75^, ^76^, ^77^, ^78^ ^]^ IAM additives are thereby thought to promote Zn deposition and inhibit parasitic processes (Figure 4g).

**Figure 5:**
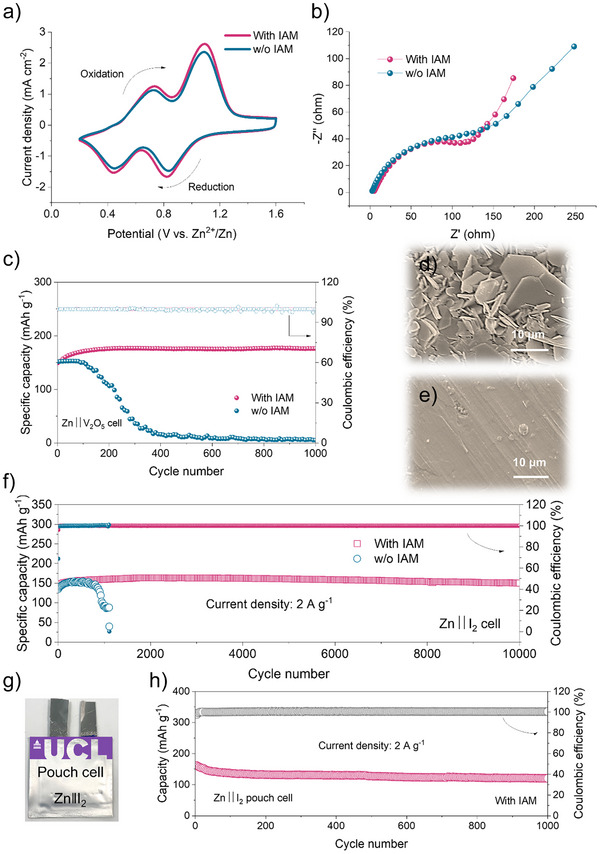
a). CV curves, b) EIS spectra, and c) cycling performance of Zn||V_2_O_5_ full cells with and without IAM additives. SEM images of cycled Zn electrodes from Zn||V_2_O_5_ full cells using electrolytes d) without and e) with IAM additives. f) Cycling performance of the Zn||I_2_ full cell at 2 A g^−1^. g) Digital photograph and h) cycling performance of the Zn||I_2_ pouch cell employing IAM additives.

In order to further assess the usefulness of IAM additives, two distinct cathodes (commercial V_2_O_5_ (Figure S9) and activated carbon supported I_2_) were combined with Zn anodes to create full cells. Generally speaking, full cell performances may be effectively improved by adding diluted IAM additives without changing cathode chemistries. The similar EIS spectra in Figure 5b and the cyclic voltammetry (CV) curves in Figure 5a support the idea that IAM additives have very little effect on the kinetics of the full cell. With IAM, the Zn||V_2_O_5_ battery demonstrated enhanced cyclability, capacity retention, and increased Coulombic efficiencies across 1000 cycles at 5 A g^−1^ at a practically low N/P ratio (the ratio of negative electrode capacity to positive electrode capacity) of 5.3, as shown in Figure 5c.

After cycling, the surface morphologies of Zn electrodes were examined to get insights into the performance enhancement of full cells. While rough, platelet‐like Zn dendrites are seen on the cycled Zn anode without IAM protection (Figure 5d), the cycled Zn anode in IAM‐containing electrolyte displays a smooth and even surface (Figure 5e), suggesting that the IAM additive allows for more uniform plating/stripping of Zn. These findings support the benefits of IAM additives once again. Remarkably, the Zn||I_2_ battery with IAM additives in Figure 5f can run steadily for 10 000 cycles at 2 A g^−1^. The Zn||I_2_ pouch cell has good cycling performance, as shown in Figures 5g,h, and S10. The aforementioned investigations corroborated the great potential of IAM additives.

## Conclusion

In this work, we present a bio‐inspired electrolyte design strategy that leverages dual‐site hydrogen bonding and *Lewis* acid–base interactions to disrupt the intrinsic symmetry of water molecules and Zn^2+^ solvation structures. By introducing isobutyramide (IAM)—a small, multifunctional molecule capable of simultaneously breaking hydrogen‐bonding networks and coordinating with Zn^2+^ ions—we effectively suppress hydrogen evolution and inhibit dendritic growth. This dual‐interaction, symmetry‐breaking approach stabilizes the electric double layer and tailors Zn^2+^ transport dynamics, enabling highly reversible and uniform Zn deposition. The resulting electrochemical performance highlights the efficacy of this strategy: Zn||Cu cells deliver a high Coulombic efficiency of 99.68% over 1000 cycles, and Zn||Zn symmetric cells achieve over 4250 hours of stable cycling without short‐circuiting. Full Zn||I_2_ cells exhibit outstanding capacity retention across 10 000 cycles, while Zn||V_2_O_5_ cells also benefit from enhanced stability—demonstrating the versatility and scalability of the IAM additive across different cathode chemistries. This study underscores the significance of symmetry disruption in electrolyte chemistry as a powerful lever to modulate solvation structures and interfacial behavior. We believe our findings will inspire the development of next‐generation aqueous electrolytes based on asymmetric molecular design and multifunctional coordination. Ultimately, this approach offers a promising path toward safe, high‐performance, and sustainable aqueous energy storage systems.

## Author Contributions

Conceptualization: W.Z. Methodology: W.Z., J.C., and C.G. Software: C.G. Validation: W.Z., J.C., and C.G. Formal analysis: W.Z. Investigation: W.Z., J.C., C.G, R.C., H.Y., and M.L. Resources: G.H. and C.G. Data curation: W.Z., J.C., and C.G. Writing—Original Draft: W.Z. Writing—Review and Editing: W.Z., J.C., C.G., and G.H. Visualization: W.Z., J.C., and T.Q. Supervision: W.Z. and G.H. Project administration: W.Z., G.H. Funding acquisition: G.H. All authors contributed to the discussions and development of this work.

## Conflict of Interests

The authors declare no conflict of interest.

## Supporting information



Supporting Information

## Data Availability

The data that support the findings of this study are available from the corresponding author upon reasonable request.
