# Supplementary material for: Harnessing Dual Hydrogen Bonding and Lewis Acid–Base Interactions for Bio‐Inspired Symmetry‐Breaking Electrolytes in Aqueous Zinc‐Ion Batteries
Source: Angew Chem Int Ed Engl. 2025 Aug 31;64(43):e202516282. doi: 10.1002/anie.202516282 (PMC12535344; doi:10.1002/anie.202516282)
Supplement: Supplementary file 1 — Supporting Information [file ANIE-64-e202516282-s001.docx]

*Supporting Information for*

Harnessing Dual Hydrogen Bonding and *Lewis* Acid-Base Interactions for Bio-Inspired Symmetry-Breaking Electrolytes in Aqueous Zinc-Ion Batteries

Wei Zhang,^a‡^* Jie Chen,^a‡^ Chaohong Guan,^b‡^ Tianyun Qiu,^c^ Xiaodong Shi,^d^ Ruwei Chen,^a^ Zhenjing Jiang,^a^ Qingjin Fu,^e^ Xian Wu,^e^ Hang Yang,^a^ Mingqiang Liu,^f^ Peie Jiang,^a^ Yunpeng Zhong,^a^ Jianbin Zhou,^g^ and Guanjie He^a^*

1. Christopher Ingold Laboratory, Department of Chemistry, University College London, London, WC1H 0AJ, UK
2. School of Materials Science and Engineering, Shanghai Jiao Tong University, Shanghai, 200240, P. R. China
3. School of Photovoltaic and Renewable Energy Engineering, University of New South Wales, Sydney, NSW 2052, Australia
4. School of Chemistry and Chemical Engineering, School of Marine Science and Engineering, State Key Laboratory of Tropic Ocean Engineering Materials and Materials Evaluation, Hainan University, Haikou, 570228, P. R. China
5. Tsinghua Shenzhen International Graduate School, Tsinghua University, Shenzhen, 518055, P. R. China
6. Max Planck Institute for Sustainable Materials, Max-Planck-Straße 1, Düsseldorf, 40237, Germany
7. Key Laboratory of Precision and Intelligent Chemistry, University of Science and Technology of China, Hefei, 230026, P. R. China

^‡^ These authors contributed equally to this work

*Corresponding author email: [wei.zhang.21@ucl.ac.uk](mailto:wei.zhang.21@ucl.ac.uk) (W. Zhang); [g.he@ucl.ac.uk](mailto:g.he@ucl.ac.uk) (G. He)

Experimental Procedures

***Electrolyte and electrode preparation*.** To synthesize the 1 M ZnSO_4_ electrolyte (BE), 0.25 mol of ZnSO_4_·7H_2_O (>99%, VWR chemicals) was meticulously dissolved in 250 mL of water (HPLC grade, Sigma-Aldrich). Subsequently, varying concentrations of isobutyramide (IAM; 99%, Sigma-Aldrich) were incorporated into the 1 M ZnSO_4_ solution, yielding final concentrations of 20 mM, 100 mM, and 500 mM. Following optimization studies, the IAM concentration was fixed at 100 mM for further investigations (DE). The I_2_@AC composite was prepared via a sublimation process, wherein equimolar amounts of iodine (I_2_; ACS reagent, Sigma-Aldrich) and activated carbon (AC; YP-80F, Kuraray Chemical) were intimately ground using an agate mortar. The resultant mixture was subjected to thermal treatment at 120 °C for 6 hours in a hydrothermal reactor, with no solvent present. V_2_O_5_ cathodes were fabricated by dispersing 70 wt.% commercial V_2_O_5_ (99.9%, Sigma-Aldrich), 20 wt.% acetylene black (battery grade, MIT), and 10 wt.% poly(vinylidene fluoride) (PVDF, battery grade, MIT) in an appropriate amount of N-methyl-2-pyrrolidone (NMP; ACS reagent, Sigma-Aldrich) solvent. Hydrophilic carbon paper (TGP-H-090, Toray) served as the current collector. The preparation method for I_2_@AC electrodes mirrored that of the V_2_O_5_ cathodes. Zinc foils have a thickness of 50 μm, unless otherwise noted.

***Materials characterizations*.** Scanning electron microscopy (SEM) images were acquired using a JEOL-JSM-6700F (Voltage: 5 kV, emission current: 110.8 µA) instrument to analyze the morphological characteristics under a low vacuum of < 3×10^-4^ Pa. The zinc plating process was monitored *in situ* with a VisiScope® BL254 T1 (VWR) microscope using a transparent Zn||Zn cell configuration at 10 mA cm^-2^. X-ray diffraction (XRD) patterns of zinc were obtained using a PANalytical Empyrean diffractometer (Cu Kα radiation; 40 kV, 40 mA) within 5~80^o^ with a step size of 0.05^o^ and a scan rate of 0.1^o^ s^-1^. Deuterium nuclear magnetic resonance (^1^H NMR) spectra were recorded on a Bruker Avance III 500 NMR spectrometer using D_2_O as the solvent. Raman spectroscopy was conducted using a LabRAM HR Evolution instrument (laser wavelength: 532 nm). Fourier-transform infrared (FT-IR) spectra were measured with a platinum-ATR FTIR spectrometer (BRUKER), and samples were analyzed in the range of 4000–400 cm^-1^ at a resolution of 4 cm^-1^, averaging 32 scans for each spectrum to enhance the signal-to-noise ratio.

***Electrochemical measurements*****.** Most electrochemical testing was conducted on CR2032 coin cells employing glass fiber membranes (Whatman GF/A). Cells were evaluated with and without IAM using a Neware battery testing system (CT-4008T-5V10mA-164 or CT-4008T-5V20mA-164, Shenzhen, China). To achieve low negative-to-positive (N/P) capacity ratios in full cells, a precise amount of zinc was electrochemically deposited on a copper substrate to match the cathode's areal capacity. The thick electrode was made by the recent method.^[1]^ Electrochemical characterizations were performed using a Biologic VMP-3 electrochemical workstation.

***DFT calculations******.*** All the density functional theory (DFT) calculations (adsorption energy and dissociation energy barrier) were performed by the generalized gradient approximation (GGA) with the exchange-correlation functional PBE.^[2]^ A cutoff energy of 400 eV was used for the plane-wave basis set. The spin geometry optimization conversions with forces on atoms and energy differences were smaller than 0.02 eV/Å and 10^-5^ Ev with a 3\times3\times1 Monkhorst-Pack k-point mesh, respectively. Moreover, the charge density differences of Zn (002)-H_2_O and Zn(002)-IAM were investigated to study the electronic structures. Besides, the electrostatic potential (ESP) and HOMO-LUMO analyses for each molecule were conducted with the B3LYP/6-31G(d,p)^[3]^ based on the Gaussian (G09) program.^[4]^

***MD simulations.*** Molecular dynamics (MD) simulations were conducted with Gromacs, version 2019.6.^[5]^ The forced field parameters were derived from opls-aa force fields.^[6]^ The SPC/E model was employed to describe H_2_O molecules. The simulation box dimensions were 9 × 9 × 9 nm^3^, with periodic boundary conditions applied in all three spatial directions. For the baseline electrolyte, the simulation cells contained 5560 H_2_O molecules and 100 ZnSO_4_ units, while for the designed system included an additional 20 IAM molecules. Electrostatic and non-electrostatic interactions were computed with a cutoff length of 1.4 nm. The integration timestep was set to 1 fs. The system was annealed from 0 to 298 K over a period of 0.5 ns, followed by a 10 ns equilibration under the NPT ensemble. A subsequent 10ns NVT MD simulation was performed for the post-equilibration analysis.

***Supplementary Figures and Tables***


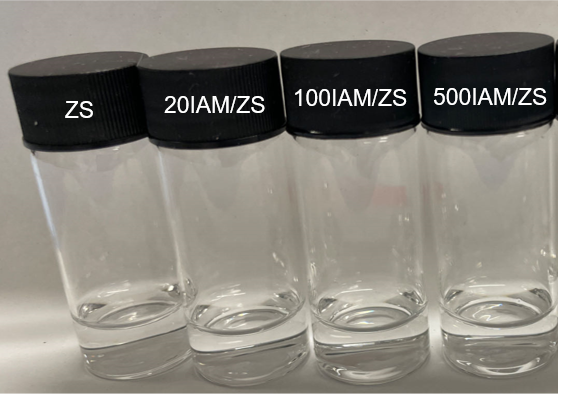


**Figure S1.** Digital photographs of the electrolytes with different concentrations of IAM.

**Figure S2.** Raman spectra of the electrolytes with/without IAM.

**Figure S3.** a) Local Raman spectra for ZnSO_4_ electrolytes with/without IAM additives. b) Two different Zn^2+^ solvation structures in IAM/ZnSO_4_ electrolyte.

**Figure S4.** Enlarged FT-IR spectra of electrolytes containing different IAM additives.

**Figure S5.** The charge distribution between IAM and H_2_O.

**Figure S6.** 3D snapshots from AIMD simulations of a) pure system without IAM and b) IAM containing system

**Figure S7.** Coulombic efficiency by Aurbach’s method.

**Figure S8.** Surface models and adsorption energies of H_2_O and IAM on different sites of
Zn (002).

**Figure S9.** XRD spectra of commercial V_2_O_5_ powders used in this work.

**Figure S10.** Charge/discharge curve of the Zn||I_2_ pouch cell with IAM additives.

**Table S1**. Comparison of the cumulative plating capacity of the ZnSO_4_-IAM-H_2_O electrolyte with recently reported Zn metal anodes with electrolyte additives optimization.

| Electrolyte  (M: mol L^-1^; m: mol kg^-1^) | Current Density (mA cm^-2^) | | Capacity (mAh cm^-2^) | Cycle Life (h) | Cumulative Capacity (mAh cm^-2^) | Ref. |
| --- | --- | --- | --- | --- | --- | --- |
| **1 M ZnSO_4_+100 mM IAM** | **10** | **1** | | **2200** | **11000** | **This work** |
| 0.5 M ZnSO_4_-7H_2_O-3DMF (H_2_O/DMF = 7:3 by volume) | 3 | 1 | | 1100 | 1650 | ^[7]^ |
| 1 M ZnSO_4_+0.5 wt % silk fibroin | 1 | 1 | | 1600 | 800 | ^[8]^ |
| 2 M ZnSO_4_+ 0.2 g L^-1^ CeCl_3_ | 2 | 1 | | 2600 | 2600 | ^[9]^ |
| 1 M ZnSO_4_+10 mM glucose | 1 | 1 | | 2000 | 1000 | ^[10]^ |
| 2 M ZnSO_4_+8 mg mL^-1^ PASP | 0.5 | 0.5 | | 3200 | 800 | ^[11]^ |
| 2 M ZnSO_4_+0.0085 M La(NO_3_)_3_ | 1 | 1 | | 1200 | 600 | ^[12]^ |
| 3 M ZnSO_4_+0.25 mM PFOA | 1 | 0.5 | | 2200 | 1100 | ^[13]^ |
| 1 M ZnSO_4_+5 vol % NMP | 1 | 1 | | 1500 | 750 | ^[3]^ |
| 1 M Zn(ClO_4_)_2_+10 mM β-CD | 1 | 1 | | 1000 | 500 | ^[14]^ |
| 3 M ZnSO_4_+10 mM TH | 5 | 1 | | 700 | 350 | ^[15]^ |
| 0.5 M Zn(CF_3_SO_3_)_2_ in DMMP and H_2_O (molar ratio 3:2) | 1 | 1 | | 2500 | 1250 | ^[16]^ |
| 1 M Zn(CF_3_SO_3_)_2_+25 mM Zn(H_2_PO_4_)_2_ | 1 | 1 | | 1200 | 600 | ^[17]^ |
| 2 M ZnSO_4_ in DMA/H2O (volumetric ratio: 2: 20) | 1 | 1 | | 1900 | 950 | ^[18]^ |

*Note: Dimethylformamide (DMF); Polyaspartic Acid (PASP); Perfluorooctanoic Acid (PFOA); N-Methyl Pyrrolidone (NMP); β-Cyclodextrin (β-CD); Threonine (TH); Dimethyl Methylphosphonate (DMMP); N, N-Dimethylacetamide (DMA).

**References**

[1] Q. Fu, W. Zhang, X. Liu, Y. Liu, Z. Lei, M. Zhang, H. Qu, X. Xiao, X. Zhong, Z. Liu, P. Qin, J. Yang, G. Zhou, *J. Am. Chem. Soc.* **2024**, *146*, 34950−34961.

[2] J. P. Perdew, K. Burke, M. Ernzerhof, *Phys. Rev. Lett.* **1996**, *77*, 3865-3868.

[3] D. Wang, D. Lv, H. Liu, S. Zhang, C. Wang, C. Wang, J. Yang, Y. Qian, *Angew. Chem. Int. Ed.* **2022**, *61*, e202212839.

[4] M. Frisch, G. Trucks, H. Schlegel, G. Scuseria, M. Robb, J. Cheeseman, G. Scalmani, V. Barone, B. Mennucci, G. Petersson, *Inc., Wallingford CT* **2009**.

[5] M. J. Abraham, T. Murtola, R. Schulz, S. Páll, J. C. Smith, B. Hess, E. Lindahl, *SoftwareX* **2015**, *1-2*, 19-25.

[6] W. L. Jorgensen, D. S. Maxwell, J. Tirado-Rives, *J. Am. Chem. Soc.* **1996**, *118*, 11225-11236.

[7] P. Zou, R. Lin, T. P. Pollard, L. Yao, E. Hu, R. Zhang, Y. He, C. Wang, W. C. West, L. Ma, O. Borodin, K. Xu, X. Q. Yang, H. L. Xin, *Nano Lett.* **2022**, *22*, 7535-7544.

[8] J. Xu, W. Lv, W. Yang, Y. Jin, Q. Jin, B. Sun, Z. Zhang, T. Wang, L. Zheng, X. Shi, B. Sun, G. Wang, *ACS Nano* **2022**, *16*, 11392−11404.

[9] Z. Hu, F. Zhang, Y. Zhao, H. Wang, Y. Huang, F. Wu, R. Chen, L. Li, *Adv. Mater.* **2022**, *34*, 2203104.

[10] P. Sun, L. Ma, W. Zhou, M. Qiu, Z. Wang, D. Chao, W. Mai, *Angew. Chem. Int. Ed.* **2021**, *60*, 18247-18255.

[11] T. Zhou, Y. Mu, L. Chen, D. Li, W. Liu, C. Yang, S. Zhang, Q. Wang, P. Jiang, G. Ge, H. Zhou, *Energy Storage Mater.* **2022**, *45*, 777-785.

[12] R. Zhao, H. Wang, H. Du, Y. Yang, Z. Gao, L. Qie, Y. Huang, *Nat. Commun.* **2022**, *13*, 3252.

[13] F. Zhao, Z. Jing, X. Guo, J. Li, H. Dong, Y. Tan, L. Liu, Y. Zhou, R. Owen, P. R. Shearing, D. J. L. Brett, G. He, I. P. Parkin, *Energy Storage Mater.* **2022**, *53*, 638-645.

[14] M. Qiu, P. Sun, Y. Wang, L. Ma, C. Zhi, W. Mai, *Angew. Chem. Int. Ed.* **2022**, *61*, e202210979.

[15] Z. Miao, Q. Liu, W. Wei, X. Zhao, M. Du, H. Li, F. Zhang, M. Hao, Z. Cui, Y. Sang, X. Wang, H. Liu, S. Wang, *Nano Energy* **2022**, *97*.

[16] S. Liu, J. P. Vongsvivut, Y. Wang, R. Zhang, F. Yang, S. Zhang, K. Davey, J. Mao, Z. Guo, *Angew. Chem. Int. Ed.* **2023**, *62*, e202215600.

[17] X. Zeng, J. Mao, J. Hao, J. Liu, S. Liu, Z. Wang, Y. Wang, S. Zhang, T. Zheng, J. Liu, P. Rao, Z. Guo, *Adv. Mater.* **2021**, *33*, 2007416.

[18] F. Wu, Y. Chen, Y. Chen, R. Yin, Y. Feng, D. Zheng, X. Xu, W. Shi, W. Liu, X. Cao, *Small* **2022**, *18*, 2202363.
